# Previous SARS‐CoV‐2 infection or a third dose of vaccine elicited cross‐variant neutralising antibodies in vaccinated solid‐organ transplant recipients

**DOI:** 10.1002/cti2.1411

**Published:** 2022-08-11

**Authors:** Chih‐Chao Chang, George Vlad, Elena Rodica Vasilescu, Ping Li, Syed A Husain, Elaine A Silvia, David J Cohen, Lloyd E Ratner, Wei‐Zen Sun, Sumit Mohan, Nicole Suciu‐Foca

**Affiliations:** ^1^ Department of Pathology and Cell Biology Columbia University Irving Medical Center New York NY USA; ^2^ Division of Nephrology, Department of Medicine Columbia University Irving Medical Center New York NY USA; ^3^ The Columbia University Renal Epidemiology (CURE) Group New York NY USA; ^4^ Department of Surgery Columbia University Irving Medical Center New York NY USA; ^5^ Department of Anesthesiology National Taiwan University Hospital Taipei Taiwan

**Keywords:** neutralising antibody, SARS‐CoV‐2, solid‐organ transplant recipient, vaccine, variant of concern

## Abstract

**Objectives:**

The SARS‐CoV‐2 pandemic poses a great threat to global health, particularly in solid organ transplant recipients (SOTRs). A 3‐dose mRNA vaccination protocol has been implemented for the majority of SOTRs, yet their immune responses are less effective compared to healthy controls (HCs).

**Methods:**

We analyzed the humoral immune responses against the vaccine strain and variants of concern (VOC), including the highly mutated‐omicron variant in 113 SOTRs, of whom 44 had recovered from COVID‐19 (recovered‐SOTRs) and 69 had not contracted the virus (COVID‐naïve). In addition, 30 HCs, 8 of whom had recovered from COVID‐19, were also studied.

**Results:**

Here, we report that three doses of the mRNA vaccine had only a modest effect in eliciting anti‐viral antibodies against all viral strains in the fully vaccinated COVID‐naive SOTRs (*n* = 47). Only 34.0% of this group of patients demonstrated both detectable anti‐RBD IgG with neutralization activities against alpha, beta, and delta variants, and only 8.5% of them showed additional omicron neutralizing capacities. In contrast, 79.5% of the recovered‐SOTRs who received two doses of vaccine demonstrated both higher anti‐RBD IgG levels and neutralizing activities against all VOC, including omicron.

**Conclusion:**

These findings illustrate a significant impact of previous infection on the development of anti‐SARS‐CoV‐2 immune responses in vaccinated SOTRs and highlight the need for alternative strategies to protect a subset of a lesser‐vaccine responsive population.

## Graphical abstract

Our findings illustrate a significant impact of previous infection on the development of anti‐COVID immune responses in vaccinated solid‐organ transplant recipients and highlight the need for alternative strategies to protect a subset of lesser‐vaccine responsive population.

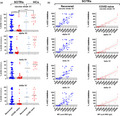

## Introduction

Solid‐organ transplant recipients (SOTRs) are at risk of severe COVID‐19, because of the use of immunosuppression and/or impaired immune defences caused by underlying diseases. The situation is further complicated by the fact that severe SARS‐CoV‐2 infections are associated with cytokine storms, an event marked by uncontrolled release of inflammatory cytokines in infected patients.^1^ Disease severity and mortality, among SOTRs with SAR‐CoV‐2, were found to be quite high in the early period of pandemic,^2^, ^3^ but have gradually trended down, as greater access to testing and improved therapeutic intervention have been implemented.^4^

Although the use of mRNA COVID vaccines provides relief for the general population by preventing severity of disease and/or contraction of the virus, the effectiveness of the standard two‐dose vaccination against the original SARS‐CoV‐2 strain has been found to be insufficient for SOTRs.^5^, ^6^ In particular, the emergence of many SARS‐CoV‐2 variants, including variants of concern (VOC), led to the authorisation of using an additional third dose of the vaccine as part of the primary immunisation series. Recent studies^7^, ^8^, ^9^, ^10^ indicate that the third dose could boost the levels of anti‐SARS‐CoV‐2 antibodies, although neutralising activities against the vaccine strain and some VOC were significantly weaker in SOTRs than in healthy controls.^7^ All the variants derive from the strain carrying the D614G mutation in the S1 region (Supplementary figure 1), which we define as the vaccine strain.

More than 80 million people in United States, including many SOTRs, contracted SARS‐CoV‐2 since March 2020. Like the general U.S. population, the majority of SOTRs who had recovered from SARS‐CoV‐2 received at least two doses of the vaccine. It is abundantly clear that vaccination increases both the levels of anti‐SARS‐CoV‐2 antibodies and the cross‐variant neutralising capacities in individuals, regardless of the status of SARS‐CoV‐2 infection.^11^, ^12^, ^13^

We previously reported that a majority of kidney transplant patients who contracted SARS‐CoV2 had retained anti‐RBD IgG, presumably the protective antibodies, but lost antinucleocapsid IgG antibodies after a prolonged period of time.^14^ In the current study, we investigated whether vaccinated SOTRs, who either recovered from COVID‐19 or had not contracted the virus (COVID‐naïve), were capable of mounting efficient humoral responses against the vaccine strain or VOC, including the current highly mutated omicron variant.

## Results

### Description of the study population

For this study, we selected a total of 113 SOTRs, including 44 recovered and 69 COVID‐naïve patients. As a control, 30 HCs, eight of whom were recovered from COVID‐19, were included. Pre‐ and postvaccination serum samples were available for all patients and controls. In the cohort, a majority of recovered‐SOTRs (33 of 44) as well as of recovered‐HCs (six of eight) contracted SARS‐CoV‐2 during the first wave of the pandemic. The time between the onset of COVID‐19 and the first vaccine dose ranged from 96 to 340 days with median 314 for patients and from 25 to 360 with median 299 for controls. As shown in Table 1, both recovered‐SOTRs (*n* = 44) and COVID‐naïve SOTRs (*n* = 69) groups shared similar demographics and clinical characteristics. Both groups were mostly kidney transplant recipients (77.3% vs 75.4%), consistent with the higher percentage of kidney transplant recipients (62.4%) in total solid‐organ transplants during the study period. They were also similar in median age (56.1 vs 61.0 years) and received organs mainly from deceased donors. In addition, most of the recipients were tested within 3‐year post‐transplantation and received similar treatments for maintenance of immunosuppression. Post‐transplant monitoring of organ recipients indicated that a vast majority (> 90%) had no donor‐specific anti‐HLA antibodies, in their sera at the time of testing. This suggests that transplanted grafts were stable at the time of vaccination and sampling as also indicated by standard clinical criteria. HCs were similar to SOTRs in age and sex, but without known comorbidities.

**Table 1:** Demographic characteristics of SOTRs and HCs

Subject characteristics	SOTRs, recovered‐	SOTRs, COVID‐naive	HCs, recovered‐	HCs, COVID‐naive
mRNA vaccine	2 doses	2 or 3 doses	2 doses	2 or 3 doses
Cases	44	69 (2 doses: 39, 3 doses: 47)	8	22 (2 doses: 17, 3 does: 14)
Ages (years) median (IQR)	56.1 (48.0–69.0)	61 (48.0–66.3)	57.8 (46–66.3)	52.2 (43–61.5)
Sex, *n* (%)				
Male	21 (47.7)	40 (58.0)	3 (37.5)	10 (45.5)
Female	23 (52.2)	29 (42.0)	5 (62.5)	12 (54.5)
Organ types, *n* (%)				
Kidney	34 (77.3)	52 (75.4)		
Heart	6 (13.6)	13 (18.8)		
Lung	4 (9.1)	7 (10.1)		
Graft types, *n* (%)				
Deceased donor	32 (72.7)	51 (73.9)		
Living donor	12 (27.3)	18 (26.1)		
Transplanted within				
0–1 year	5	12		
1–3 years	26	36		
3–5 years	10	14		
> 5 years	3	7		
Donor‐specific antibodies (DSA) on serum samples, *n* (%)				
Class I only	1 (2.2)	1 (1.4)		
Class II only	1 (2.2)	3 (4.3)		
Class I & class II	1 (2.2)	3 (4.3)		
Immunosuppression regimen, *n* (%)				
Prednisone	26 (55.3)	40 (58.0)		
Calcineurin inhibitors	28 (59.5)	52 (75.4)		
mTOR inhibitors	3 (6.4)	5 (7.2)		
Antimetabolites	33 (70.1)	49 (77.8)		
Belatacept	7 (14.9)	3 (4.3)		
Time between COVID onset and 1st vaccine, median (IQR)	314 (96–340)	–	299 (25–360)	–
Time between 2nd vaccine and serum sample, median (IQR)	93 (41–138)	81 (47.5–105.5)	141 (102–168)	185 (172.5–200.5)
Time between 3rd vaccine and serum sample, median (IQR)	–	56 (35–90)	–	27 (23–36)

Within the 69 COVID‐naïve SOTRs cohort, 22 subjects had specimens available only for the two‐dose study, and 30 has specimens available only for the three‐dose study, whereas 17 had specimens available for both two‐dose and three‐dose studies. In the COVID‐naïve HCs series, eight subjects had specimen available only for two‐dose study and five has specimens available only for the three‐dose study, whereas nine had specimens available for both post‐ two doses and post‐ three doses testing. Three patients had two organs transplanted. Time (days) between the events and ages (years) were presented as median (interquartile range).

### Extreme heterogeneity of anti‐SARS‐CoV‐2 immune response in vaccinated SOTRs

**Figure 1:**
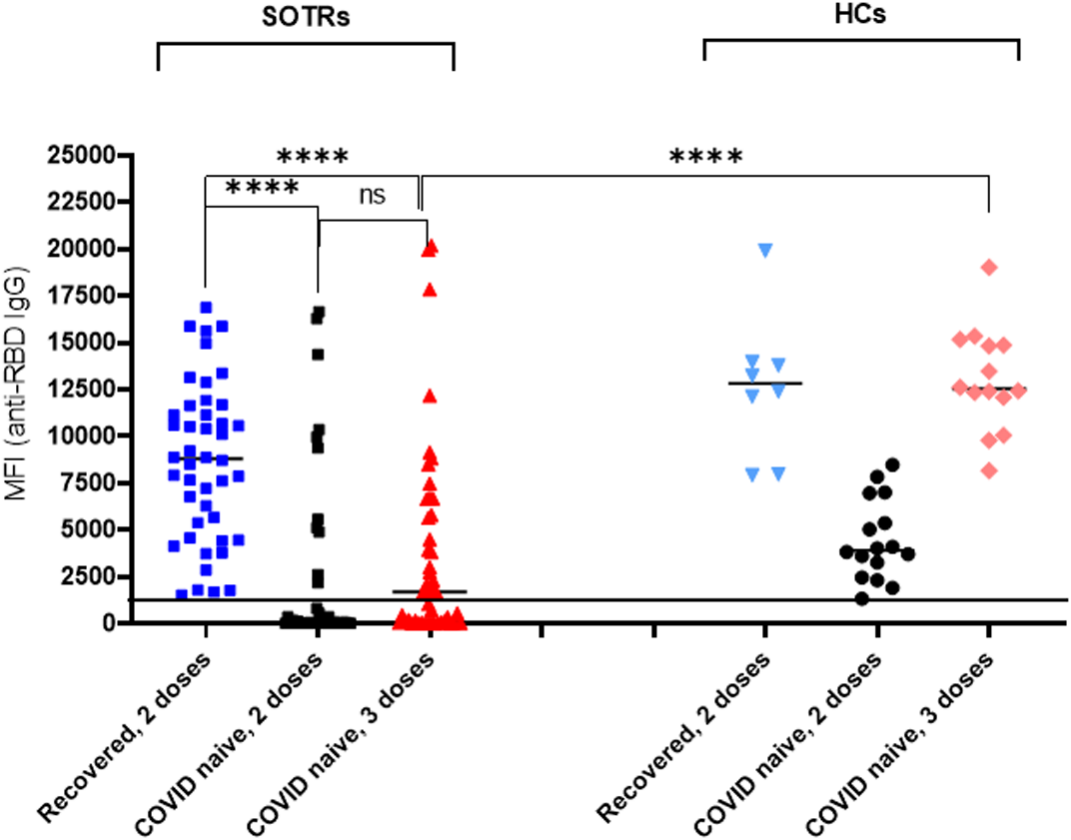
Vaccination‐induced anti‐RBD IgG antibodies in SOTRs and HCs. Sera from recovered‐SOTR (*n* = 44) who received two doses of vaccines and sera from COVID‐naïve (*n* = 69) who received 2 (*n* = 39) or three doses (*n* = 47) of vaccines, along with samples from recovered‐HCs (*n* = 8) and COVID‐naive HCs (*n* = 22) were tested for IgG antibodies against SARS‐CoV‐2 antigens (RBD, S1 and nucleocapsid) by multiplexed magnetic bead‐based assay. Levels of antinucleocapsid IgG antibodies were used for confirmation of COVID infection. Positivity (> 700 MFI) of assay, pre‐et by manufacturer, is denoted as a horizonal line. **** was referred to *P* < 0.0001.

The levels of anti‐SARS‐CoV‐2 (nucleocapsid, RBD and S1) IgG antibodies in postvaccine sera collected from of SOTRs and HCs were analysed (Figure 1). We excluded testing of anti‐SARS‐CoV‐2 IgM since sera were collected 30–185 days after vaccination or disease onset, a time when testing of IgM antibodies to SARS‐CoV‐2 is unlikely to be of diagnostic or prognostic value.^15^ We found that the production of anti‐RBD IgG antibodies in COVID‐naive SOTRs following vaccination was extremely heterogeneous, than in their HC counterparts. While HCs were 100% seropositive post‐two‐dose and post‐three‐dose vaccination, COVID‐naive SOTRs were 28.2% (11/39) seropositive 81 days after two‐dose vaccination, and 55.3% (26 of 47) seropositive 56 days after three‐dose vaccination. In addition, while the median antibody levels for COVID‐naïve HCs was 3920 (2668–6568) MFI post‐two‐dose vaccination and 12 532 (11 583–14 956) and MFI post‐three‐dose vaccination, the median antibody level for COVID‐naive SOTRs was 84 (30–2170) MFI post‐two‐dose vaccinations and 1699 (86–5806) MFI post‐three‐dose vaccination, respectively. The patterns of anti‐S1 IgG levels in these patients were similar but were about ½–¼ of that of anti‐RBD IgG (data not shown).

These results reveal the very weak and heterogeneous nature of anti‐SARS‐CoV‐2 immune responses in COVID‐naïve SOTRs after vaccination. There was an increase in the levels and the percentages of anti‐RBD IgG seropositivity in COVID‐naïve SOTRs after the third dose of vaccination. This was not significantly higher (*P* = 0.265) than after the second dose; it did, however, remain significantly lower (*P* < 0.0001) than in COVID‐naïve HCs who received three‐dose vaccination.

Analyses of anti‐RBD IgG in vaccinated recovered‐SOTRs (*n* = 44) revealed that these patients mounted a significantly higher immune response against SARS‐CoV‐2. All 44 patients were anti‐RBD seropositive. The median level of IgG antibodies, (8797 MFI), was significantly higher than that of COVID‐naïve patients who received either two‐dose (*P* < 0.0001) or three‐dose (*P* < 0.0001) vaccinations.

### Differential neutralisation capacities between vaccinated recovered‐ and vaccinated COVID‐naïve SOTRs

To determine whether sera from vaccinated SOTRs can neutralise the SARS‐COV2 vaccine strain and VOC, we chose two different approaches. The first was based on the ability of sera to prevent the entry of SARS‐CoV‐2 pseudotyped lentiviral particles into cells overexpressing the ACE2 receptor protein, 293‐ACE2 cells. The second was to test the ability of sera to inhibit binding of probed viral Spike proteins to ACE2. For the latter, we used a commercially available multiplexed neutralisation system, which allows the simultaneous determination of the neutralising capacities of sera against the vaccine strain and SARS‐CoV‐2 (alpha, beta and delta) variants (Supplementary figure 1). As shown in Supplementary figure 2, we demonstrated that both methods could accurately identify the neutralisation capacities against the vaccine strain and alpha, beta and delta variant strains on a wide range of clinical diagnostic sera samples. Although the multiplexed ACE2 inhibition assay is 10‐ to 20‐fold weaker in sensitivity, it is highly desirable for clinical testing because of its effectiveness as a high‐throughput assay.

**Figure 2:**
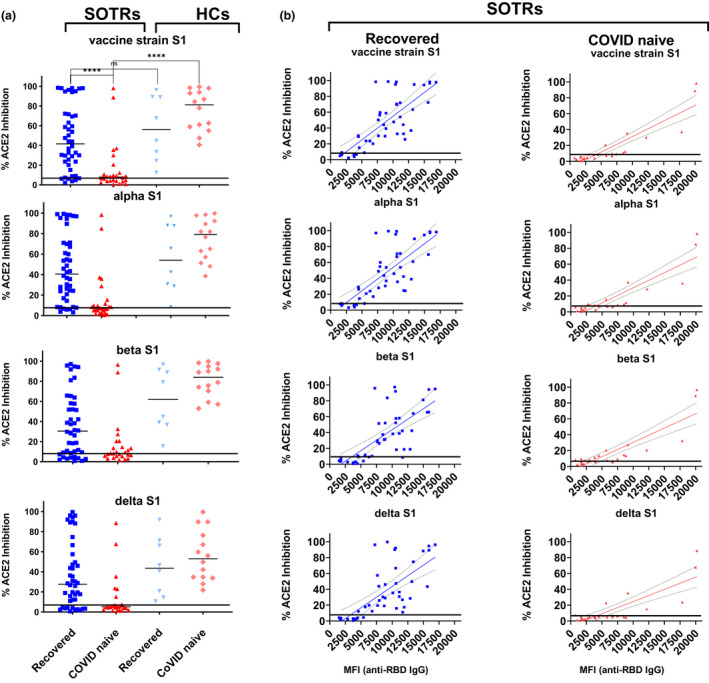
Neutralising capacities of sera from vaccinated SOTRs or HCs against the vaccine and alpha, beta and delta variants. **(a)** Multiplexed neutralisation assays were used to determine the degree of inhibition (%) by sera on binding of indicated viral S1 proteins to ACE2 receptor. A horizonal line (8% inhibition at 1:200‐fold dilution) representing the positive neutralising cut‐off was derived from the results of Supplementary figure 2. The qualitative analysis (% inhibition) is defined as 100 × (1 − sample value /negative control value). **(b)** Linear regression analyses were carried out to determine relationships between the levels of anti‐RBD IgG antibodies (*X* axis) and % ACE2 inhibition (*Y* axis). In the recovered‐SOTRs series, levels of anti‐RBD IgG were found strongly correlated with the degree (%) of inhibition on binding of vaccine strain (*R* ^2^ = 0.60; *P* < 0.0001), alpha (*R* ^2^ = 0.59; *P* < 0.0001), beta (*R* ^2^ = 0.51; *P* < 0.0001) and delta (*R* ^2^ = 0.48; *P* < 0.0001) to ACE2. In the COVID‐naïve SOTRs series, the correlations between these two events were even stronger, with vaccine strain (*R* ^2^ = 0.80; *P* < 0.0001), alpha (*R* ^2^ = 0.79; *P* < 0.0001), beta (*R* ^2^ = 0.75; *P* < 0.0001) and delta (*R* ^2^ = 0.69; *P* < 0.0001), respectively. **** was referred to *P* < 0.0001.

To determine whether sera from subjects positive for anti‐RBD IgG could have neutralising capacities against the SARS‐COV‐2 vaccine strain and VOC variants, samples from either fully vaccinated (three‐dose) COVID‐naïve SOTRs, who were anti‐RBD IgG^+^ (*n* = 26) or partially (two‐dose) vaccinated recovered‐SOTR (*n* = 44), along with the counterparts from HCs, were measured for their abilities to inhibit viral S1‐ACE2 binding. As shown in the Figure 2a, 79.5% (35 of 44) of recovered‐SOTRs and 61.5% (16 of 26) of COVID‐naïve SOTRs showed detectable neutralising activities. If patients with seronegative sera were included, only 34.0% (16 of 47) of COVID‐naïve SOTRs displayed immune responses against SARS‐CoV‐2 after three doses of vaccination. Median inhibition capacities of sera from recovered‐SOTRs ranged from 27.7% to 41.6% against four different viral S1s. These were significantly higher (*P* < 0.0001) than the results from COVID‐naïve‐SOTRs, which ranged from 5.1% to 8.0%, but were not significantly different from their HC counterparts. Among S1 proteins, the most resistant to neutralisation by sera was the delta variant, with a median of 27.7% inhibition for recovered‐SOTRs and a median of 5.1% inhibition for COVID‐naïve SOTRs. Sera from both HC groups, however, showed median > 44% cross‐strain neutralisation capacities.

To further characterise the neutralisation patterns occurring in vaccinated recovered‐SOTRs and fully vaccinated COVID‐naïve SOTRs, we directly compared sera neutralisation activities of these two groups of patients against levels of anti‐RBD IgG (Figure 2b). We found that neutralisation activities against all four viral strains were strongly positive‐correlated (*P* < 0.0001) with levels of anti‐RBD IgG antibodies in both recovered‐SOTRs and COVID‐naïve SOTRs. Spearman *r* ranged from 0.762 to 0.726 for recovered‐SOTRs, and from 0.874 to 0.675 for COVID‐naïve SOTRs. Our findings suggest that impairment in neutralising viruses in COVID‐naïve SOTRs was because ofthe inability to produce anti‐RBD IgG antibodies, and that boosting antiviral RBD IgG levels may increase the cross‐neutralising effect against the vaccine strain and VOC.

### Significant escape from neutralisation by the omicron variant in fully vaccinated COVID‐naïve SOTRs

**Figure 3:**
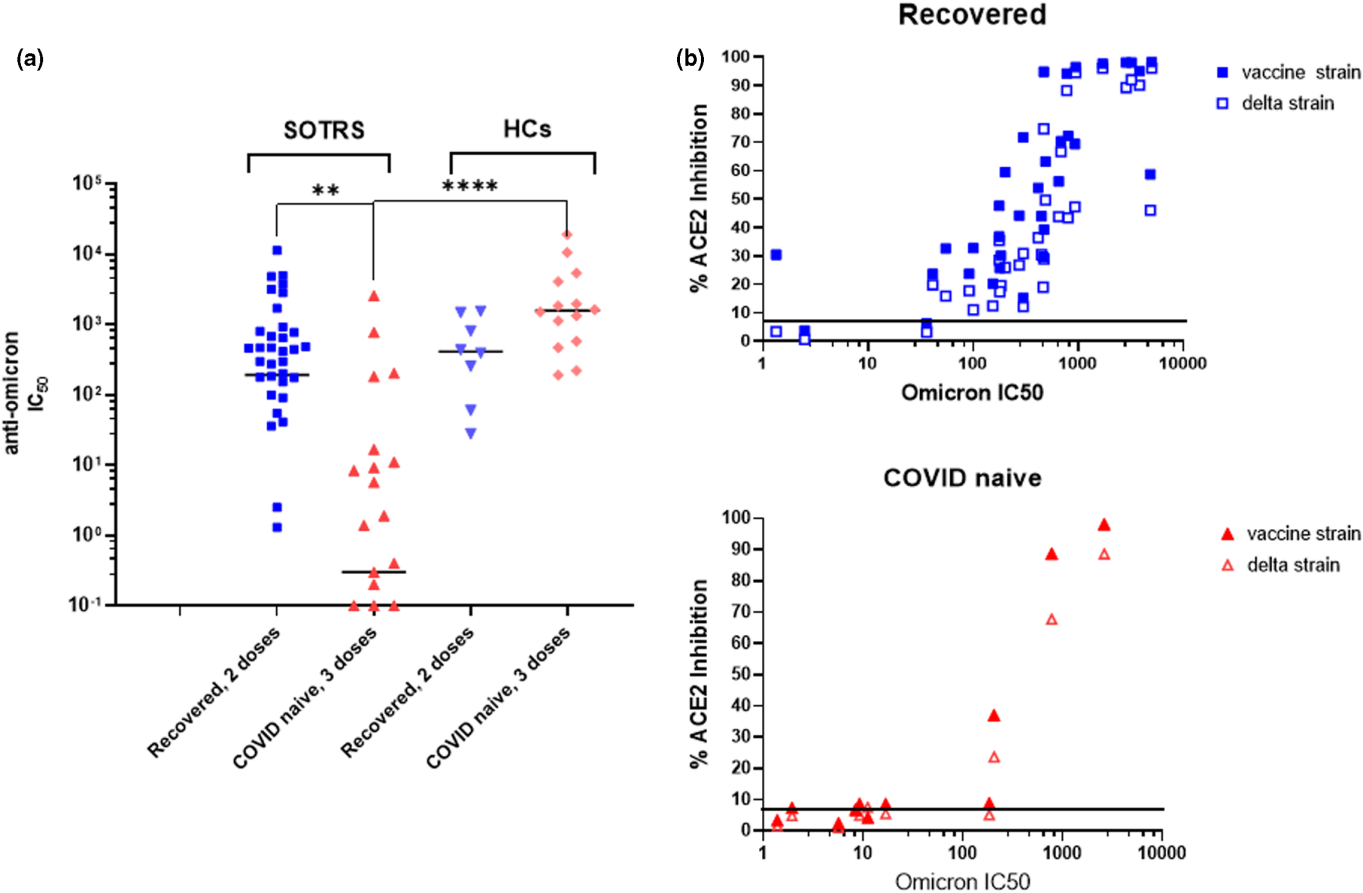
Neutralising capacities of sera from vaccinated SOTRs and HCs against omicron. **(a)** Omicron‐neutralisation of COVID‐naïve SOTRs (median IC_50_ = 0.3) was significantly lower than that of recovered‐SOTRs (median IC_50_ = 193, *P* = 0.0027) or COVID‐naïve HCs (median IC_50_ = 414, *P* < 0.0001). **(b)** Correlation tests between omicron‐neutralisation with vaccine‐ and delta‐neutralisation. In the recovered‐SOTRs series, omicron‐neutralisation strongly correlated with both vaccine strain‐ (Spearman *r* = 0.870, *P* < 0.0001) and delta strain‐neutralisation (*r* = 0.869, *P* < 0.0001). In the COVID‐naïve SOTRs series, omicron‐neutralisation was modestly correlated with both vaccine strain‐ neutralisation (Spearman *r* = 0.326, *P* = 0.104) and delta strain‐neutralisation (Spearman *r* = 0.376, *P* = 0.058). ** was referred to *P* = 0.0027 and **** was referred to *P* < 0.0001.

To test whether SOTRs positive for anti‐RBD IgG antibodies were protective against highly mutated VOC such as omicron, we tested the ability of their sera, as well as of sera from the HCs, to neutralise the omicron variant in pseudotyped neutralisation assays. As shown in Figure 3a, we found that fully vaccinated COVID‐naïve SOTR displayed low and extremely heterogeneous immune responses against omicron. The median level of neutralising activities (IC_50_ = 0.3) in this group of individuals was significantly lower than that of fully vaccinated COVID‐naïve HCs (IC_50_ = 1582, *P* < 0.0001). Only four of 47 of COVID‐naïve SOTRs showed neutralising activity on par with those of HCs. In contrast, vaccinated recovered‐SOTR demonstrated higher neutralisation activity against omicron. Their neutralising antibody titres (median IC_50_ = 193) were not significantly different from those of vaccinated recovered‐HC (median IC_50_ = 414, *P* = 0.404) or of fully vaccinated naïve‐HCs (IC_50_ = 1582, *P* = 0.088), but were significantly higher (*P* = 0.0027) than those of fully vaccinated COVID‐naive SOTRs. To better understand the omicron‐neutralisation patterns observed in sera from these two groups of patients (vaccinated COVID‐naïve and vaccinated recovered), we compared their omicron‐neutralisation with neutralisations against the vaccine strain and the delta variant. We demonstrated that omicron‐neutralisation was strongly correlated with neutralisation against the vaccine strain (Spearman *r* = 0.870, *P* < 0.0001) and against delta variant (Spearman *r* = 0.869, *P* < 0.0001) in the vaccinated recovered‐SOTRs but were weakly correlated with the neutralisation against the vaccine strain (Spearman *r* = 0.326, *P* = 0.104) and the delta variant (Spearman *r* = 0.376, *P* = 0.058) in the vaccinated COVID‐naïve SOTRs (Figure 3b).

## Discussion

Our results suggest that previous SARS‐CoV‐2 infection not only triggers the induction of higher titres of neutralising antibodies in SOTRs against both the vaccine strain and lesser‐mutated delta variant but raises the breadth of overall humoral immunity reflected in cross‐reactivities, allowing efficient neutralisation against omicron, a highly mutated variant. A vast majority of fully vaccinated COVID‐naïve SOTRs, on the contrary, failed to do so. Since a meta‐analysis of 100 000 cases from 24 studies^16^ showed that neutralising antibodies are predictive of protection against SARS‐CoV‐2, we analysed neutralising antibodies in SOTRs. Our study provides some important insight into the interplay between naturally acquired and vaccine‐induced antiviral immune responses in SOTRs.

Compared with HCs and recovered SOTRs, humoral responses against COVID‐19 vaccine in the COVID‐naïve group were low and very heterogeneous. Very few of COVID‐naïve SOTRs produced levels of omicron‐neutralising antibodies comparable with those from HCs. Omicron variants have been shown to be well‐equipped to escape neutralisation by many therapeutic monoclonal antibodies, yet retain a high affinity for the ACE2 receptor.^17^, ^18^ Our study is consistent with similar investigations,^19^, ^20^, ^21^, ^22^ which demonstrated that vaccine‐induced cross‐neutralisation against omicron was significantly decreased in dialysis or post‐transplant patients.

Importantly, we found that the majority of recovered‐SOTRs maintained a robust antiviral response, with respect to levels of anti‐RBD antibodies and cross‐variant neutralising capacities, at levels almost on par with those found in HCs. We suggest that naturally acquired anti‐SARS‐CoV‐2 immune responses via infection, even if mild, were more stimulatory than the vaccine‐induced immunities, as also demonstrated by others.^23^ Our data also support the conclusion of a large cohort study^24^ showing that vaccinated‐recovered individuals have a slower waning, longer‐lasting anti‐SARS‐CoV‐2 immunity than either vaccinated COVID‐naive or unvaccinated recovered individuals. The effectiveness of T cell‐dependent antibody responses is optimised by preferentially steering B cells reactive against high affinity or abundant epitopes towards plasma cell differentiation.^25^ Recent studies indicated that being immunosuppressed, transplant patients present deeply blunted anti‐SARS‐CoV‐2's RBD‐specific memory B cells and neutralising antibody response caused by the reduced frequency of SARS‐CoV‐2 specific T cells within the germinal centres.^26^, ^27^ Processing and presentation of multiple HLA‐bound viral epitopes by macrophages and dendritic cells may contribute to intra‐ and intermolecular spreading of cryptic epitopes. This may explain the broad spectrum of antivariant antibodies in COVID‐19‐recovered patients.^28^ Recent studies show that impaired cellular immune responses against COVID‐19 are related to the differentiation of myeloid‐derived suppressor cells, resulting in the differentiation of regulatory T cells.^29^ We suggest that depletion of the suppressor cell population and/or cross‐linking of activating receptors on the membrane of primed T cells may enhance patients' immune responses to COVID‐19.

## Methods

### Human subjects, patient privacy protection

This retrospective study was approved by a protocol (AAAT3602) from the Institutional Review Board at Columbia University Irving Medical Center (CUIMC). In all, a total of 116 solid‐organ transplants in 113 recipients (SOTRs), who returned to CUIMC for patient care from March 2020 to January 2022 and had been vaccinated with 2–3 doses of SARS‐CoV‐2 mRNA vaccines (either Moderna mRNA‐1273 or Pfizer‐BNT 162b2) were identified through our medical record system. COVID‐19 diagnosis was confirmed by a positive SARS‐CoV‐2's RT‐PCR test on nasal swab samples. The study population composed of 86 kidneys, 19 heart and 11 lung transplant recipients. Three recipients had undergone a double transplant. The number of patients with various transplants largely corresponds to the percentage of kidney (64), heart (13) and lung (24) transplants from the 1200 transplants performed during the study period. At CUIMC, immunosuppression reduction in kidney transplant recipients diagnosed with COVID‐19 was based on severity of symptoms and intensity of pre‐infection immunosuppression. For patients on immunosuppression regimens including antimetabolites (e.g. azathioprine and mycophenolate), the antimetabolite dose reduced or held depending on severity of symptoms and clinician‐determined risk of immunosuppression reduction. For patients on calcineurin inhibitors alone or with a corticosteroid only, no change in immunosuppression was recommended. Immunosuppression was returned to pre‐infection baseline approximately 2 weeks after improvement in the acute infection. Also included were 30 healthy controls (HCs), either healthcare workers or volunteers. Verbal informed consent was given by HCs.

### Laboratory finding and sample collection

Authorisation for use of de‐identified SOTRs specimens from clinical care that would otherwise be discarded and use of de‐identified laboratory results of SOTRs were approved by a waiver (AAAP2200) through HIPPA Guidelines. Serum samples of SOTRs, previously collected for patient care, were identified by Histotrac Software (One Lambda, West Hills, CA, USA) from the Serum Bank of the Immunogenetics Laboratory of CUIMC. Sera from HCs were prepared from whole blood via centrifugation, aliquoted, and frozen at −20°C.

### Plasmids, cell lines, transfections and SARS‐CoV‐2 pseudoviral particles production

The following plasmids were obtained from Addgene.org (Watertown, MA, USA): pcDNA3.3_CoV2_B.1.1.7 (alpha variant, Plasmid#170451), pcDNA3.3_CoV2_501V2 (1.351, beta variant, Plasmid #170449), pcDNA3.3‐SARS2‐B.1.617.2 (delta variant, Plasmid #172320) and pTwist‐SARS‐CoV‐2 ∆18 B.1.1.529 (Omicron variant, Plasmid #179907). All these plasmids encode spike proteins with c‐terminal 18 aa deletion. The following plasmids were obtained from BeiResources.org (Manassas, VA, USA): pHDM‐SARS‐CoV‐2Spike D614G (NR‐53765), pHAGE‐CMV‐Luc2‐IRES‐ZsGreen‐W (NR‐52516), pHDM‐Hgpm2 (NR‐52517), pHDM‐tatt11b (NR‐52518) and pRCCMV‐rev1b (NR‐52519). The human ACE2 stably transfected cell line, 293T‐ACE2, was also from Bei Resources.

SARS‐CoV‐2's pseudoviral particles were prepared from transfection of 293T cells with lentiviral packing plasmids, together with various SARS‐CoV‐2 Spike coding plasmids, according to a method described by Crawford *et al*.^30^ Plasmid DNA was purified by the Qiagen EndoFree® (Germantown, WI, USA) plasmid kit; transfections were performed with lipofectamine® 3000 (Fisher Scientific, PA, USA). All SARS‐CoV‐2 pseudotyped lentiviral particles were filtered through a 0.45‐μm filter, concentrated by centrifugation through a 10% sucrose cushion, aliquoted, and stored at −80°C.

### SARS‐CoV‐2 pseudotyped neutralisation assays

Neutralisation assays were performed by incubating SARS‐CoV‐2 pseudovirus with serial dilutions (1:10 to 1:10 000) of sera and scored by the reduction in luciferase gene expression. In brief, 25 μL pseudovirus (3–5 × 10^5^ RLU) were incubated with an equal volume of diluted sera in a 96‐well plates at 37°C for 45 min; 40 μL of serum/virus mix was then transferred to wells of a cell‐culture 96‐well plates. Each well was preseeded with 1.5 × 10^4^ 293T‐ACE2 cells in 60 μL medium (DMEM supplemented with 10% FCS) in the presence of 8 μg mL^−1^ polybrene. After 60‐h postinfection, cells were collected for luciferase assays. The Promega (Madison, WI, USA) BrightGlo® Luciferase Assay System with Promega GloMax® Plate Reader was used for detection of luciferase activity. The qualitative analysis (% neutralisation) is defined as 100 × (1 − (sample's RLU − background's RLU)/(positive control's RLU negative control's RLU)). IC_50_ (half maximum inhibitory concentration) values were calculated using non‐linear regression in GraphPad Prism 9.2.0 (San Diego, CA, USA).

### Multiplexed magnetic bead‐based assay for detection IgG antibodies against SARS‐CoV‐2's viral antigens

The xMAP® SARS‐CoV‐2 Multi‐Antigen IgG system (Luminex, Austin, TX, USA) for simultaneous detection of viral targets (RBD, S1 and nucleocapsid viral proteins) was previously described.^14^ The positivity of anti‐viral IgG antibodies was preset by the manufacturer as equal to or above 700 MFI.

### Multiplexed magnetic bead‐based assay for detection of neutralising antibodies against the vaccine strain or VOC

Pro® Human SARS‐CoV‐2 Neutralisation Assay (Bio‐Rad, Hercules, CA, USA) was used for detection of neutralisation activities with some modifications. In brief, multiplexed magnetic beads were prepared by mixing SARS‐CoV‐2 neutralisation antibody 2‐Plex (the original Spike 1 and RBD) with viral antigen coupled beads of αS1, βS1, and δ Spike Trimer (all from Bio‐Rad). After the second wash, magnetic beads were mixed with 25 μL of serially diluted (1:5 to 1:1500) subjects' sera and incubated at room temperature (RT) for 30 min with shaking. Biotin‐labelled human ACE2 (25 μL) was then added to the reaction wells and incubated at RT for another 30 min. After three washes, ACE2 binding magnetic beads were incubated with 50 μL detection reagent (streptavidin‐PE) at RT for 10 min with shaking. Resuspended beads were transferred to a V‐bottom 96‐well plates and run on a Luminex®200 Platform. Data (MFI) were acquired using xPONENT® Software and analysed with Microsoft's Excel Software. The manufacturer's cut‐off for positive neutralisation was the percentage of inhibition above 10% with sera diluted at 1:5. Based on the results from Supplementary figure 2, our positive neutralisation cut‐off was set to equal or above 8% inhibition with sera diluted at 1:200 fold, or equal or above 30% inhibition with sera diluted at 1:20.

### Statistics

Statistical analyses and generation of the graphs were carried out using GraphPad Prism 9.2.0. An unpaired *t*‐test with Welch's correction was used to compare two groups of variables. The Spearman correlation coefficient *r* was calculated for quantifying the association between continuous variables. Two‐tailed *P*‐values were reported, with *P* < 0.05 considered to be significant.

## Conflict of interest

The authors declare no conflict of interest.

## Author contributions

**Chih‐Chao Chang**: Conceptualization; Data analysis; Fund acquisition; Project administration; Writing – original draft and editing and reviewing. **George Vlad** and **Elena Rodica Vasilescu**: Funding acquisition; Data analysis. **Ping Li**: Methodology; Editing. **Elaine A Silvia**: Project administration; Editing. **Wei‐Zen Sun**: Conceptualization. **David J Cohen** and **Lloyd E Ratner**: Writing – review and editing. **Syed A Husain** and **Sumit Mohan**: Data curation, Investigation. **Nicole Suciu‐Foca**: Conceptualization; Data curation; Writing – review and editing.

## Supporting information


Supplementary figures 1 and 2
