# Supplementary material for: Previous SARS‐CoV‐2 infection or a third dose of vaccine elicited cross‐variant neutralising antibodies in vaccinated solid‐organ transplant recipients
Source: Clin Transl Immunology. 2022 Aug 11;11(8):e1411. doi: 10.1002/cti2.1411 (PMC9371857; doi:10.1002/cti2.1411)
Supplement: Supplementary file 1 — Supplementary figures 1 and 2 [file CTI2-11-0-s001.docx]

**
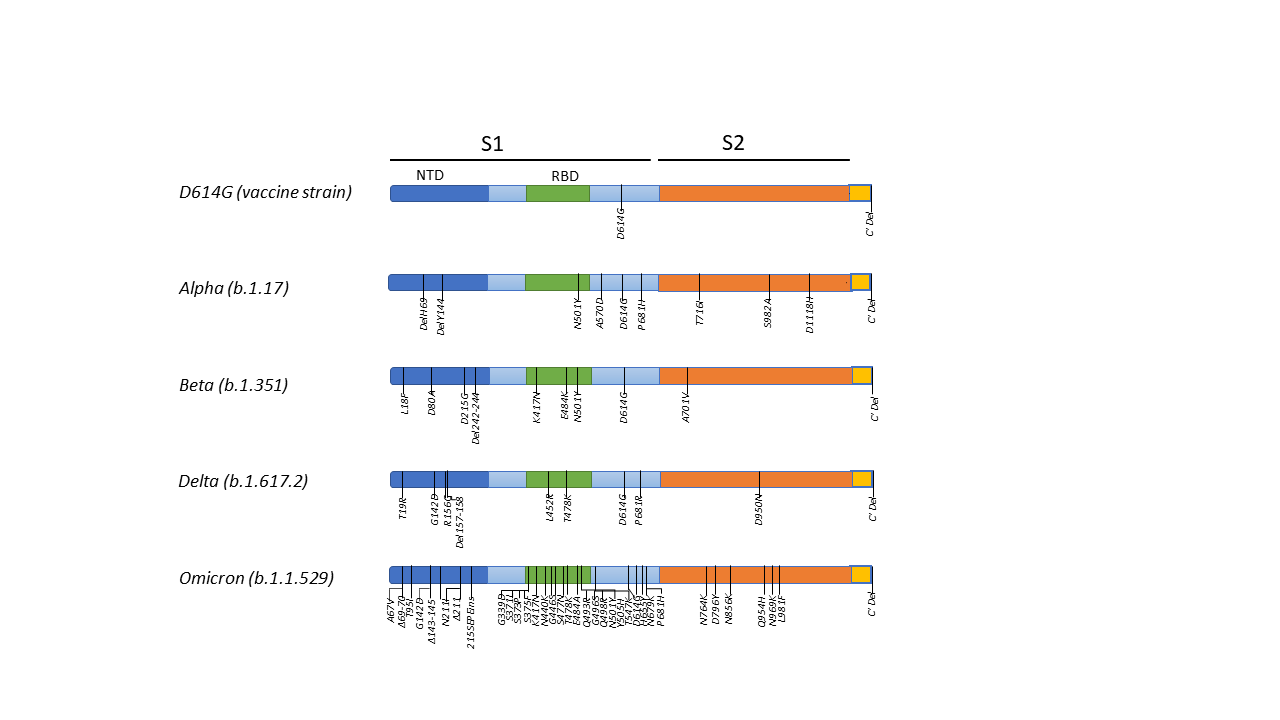
**

**Supplementary figure 1. Diagram illustrating the spike(S) proteins of the vaccine strain, α, β, δ, and O variants**. Mutations present in each of the spike protein variants are labeled. Since all known variants carrying the D614G mutation in the non- receptor binding domain (RBD) region of S1 subunit, the D614G strain was used as the representative of the vaccine strain.

**
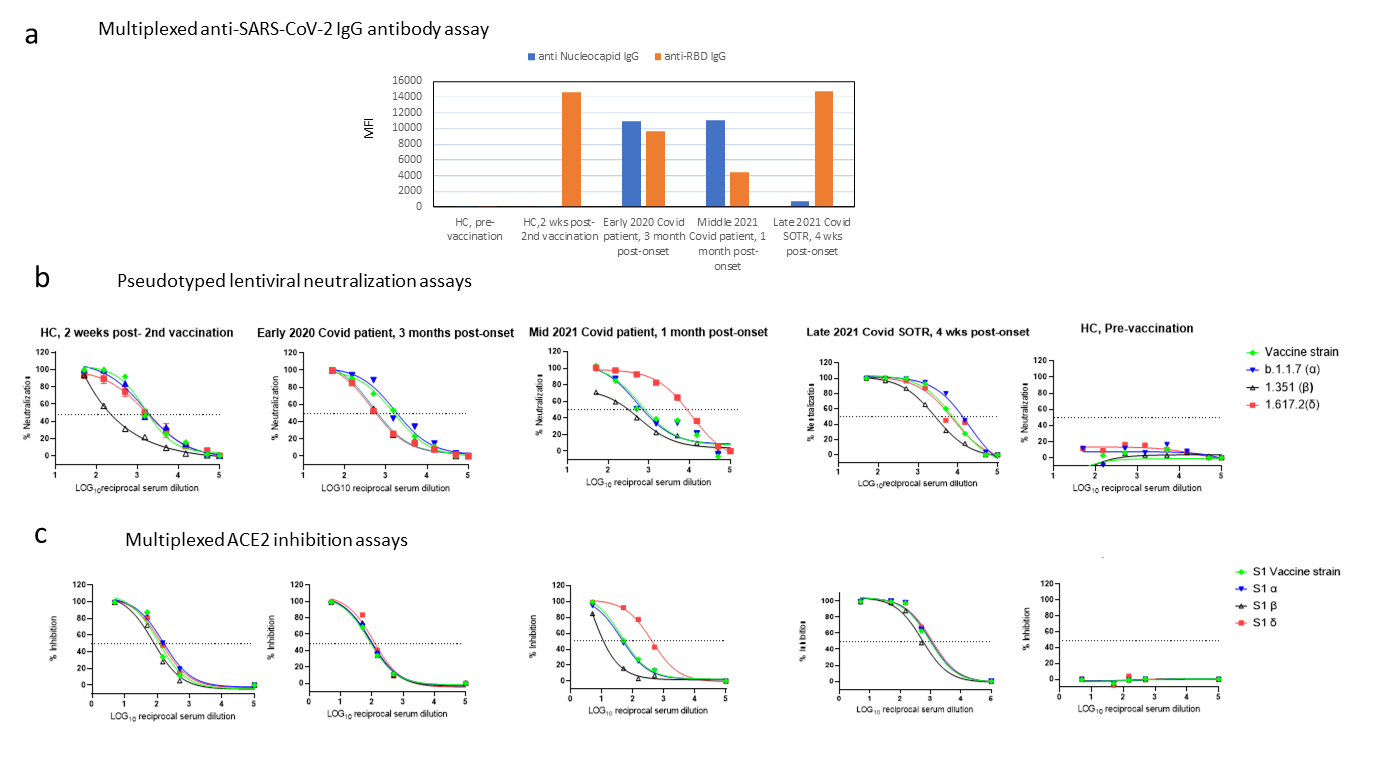
**

**Supplementary figure 2. Detection of neutralizing activities in vaccinated or SARS-CoV-2 infected individuals.** A panel of diagnostic serum samples (two samples, pre-vaccinated and post 2 doses vaccinated, from a healthy control; one from an early 2020 COVID-19 patient; one from a mid-2021 COVID-19 patient and one from a late 2021 breakthrough-COVID SOTR) were measured for anti-SARS-CoV-2 IgG, pseudotyped neutralization assays and multiplexed ACE2 inhibition assays. **(a)** Profiles of anti-SARS-CoV-2 IgG antibodies in the diagnostic samples. The presence of anti-RBD and S1 was used to monitor both anti-viral responses and vaccine efficacies, the presence of anti-nucleocapsid antibodies was used as confirmation of previous SARS-CoV-2 infection. Individuals with breakthrough-infection had high levels of anti-RBD and S1 IgG but low level of anti-nucleocapsid IgG. **(b)** SARS-CoV-2 pseudotyped neutralization assays were performed with a serially (50–50,000-fold) diluted serum. The sample from late 2021 breakthrough-COVID SOTR had the highest overall IC_50_ values, ranging from highest 12,312 against alpha S1 to lowest 2,617 against beta S1, whereas the sample from mid- 2021 COVID-19 subject showed a skewed anti-viral immune response, with strongest activities (IC_50_ = 7861) against the delta variant and weakest activities (IC_50_ = 253) against the beta variant. This typical immune response has been described in the other studies^13^. **(c)** ACE2 inhibition assays performed with a serially (5 - 5,000) diluted sera used in the Panel **b**. Average median IC_50_ against all 4 viral S1 proteins for all samples was 114.5 (79.9 -457). Similar to Panel **b**, ACE2 inhibition assays demonstrated that serum from the breakthrough-SOTR had the highest antiviral immune activities and serum from mid- 2021 COVID subject had a skewed anti-viral immune response. Based on this result, we diluted majorities of sera 200-fold for this assay. No pre-existing anti-SARS-CoV-2 IgG, or neutralizing activities were found in serum from pre-vaccinated individuals.
